# Physicians in Training Learning Endoscopy: Reduction in Radiation Exposure with Optical Navigation Technology

**DOI:** 10.3390/jcm15072579

**Published:** 2026-03-27

**Authors:** Audrey Demand, Samuel B. Kankam, Chris Oake, Paul Holman, Meng Huang

**Affiliations:** 1 Department of Neurological Surgery, Houston Methodist Hospital, Houston, TX 77030, USA; sbkankam@houstonmethodist.org (S.B.K.); jcoake13@tamu.edu (C.O.); pjholman@houstonmethodist.org (P.H.); mhuang@houstonmethodist.org (M.H.); 2 T.H. Chan School of Public Health, Harvard University, Cambridge, MA 02115, USA; 3 Texas A&M School of Engineering Medicine, Houston, TX 77030, USA

**Keywords:** endoscopic spine surgery, radiation, 2D Fluoroscopic navigation, minimally invasive spine surgery

## Abstract

**Background:** The endoscopic approach to the spine has a steep learning curve, primarily due to challenges in learning how to access Kambin’s triangle, leading to significant radiation exposure. Real-time live instrument tracking using two-dimensional fluoroscopic navigation has been shown to significantly reduce the time and radiation exposure needed to perform a trans-Kambin approach; however, the impact on the learning curve for those in training has not been studied. **Methods:** Physicians in training (PITs) in a single program were evaluated while accessing Kambin’s triangle using a gel sawbone model. The PITs were randomized in terms of spinal level, the technology used first, and the approach side. Time to access Kambin’s triangle and radiation were recorded for each cohort. A linear regression model was used to assess associations between procedure time and radiation exposure and endoscopic experience and PGY level. **Results:** Seven PITs were studied with a range of prior experience, consisting of three PITs with high exposure (HE) (≥30 cases) and four with low exposure (LE) (0–20 cases, averaging 7.5). Unassisted by instrument tracking, the SE group was 63% faster and was exposed to 63% less radiation. Further, there was a moderate correlation with experience and time (R = −0.52) and radiation (R = −0.60). Using instrument tracking allowed all the PITs to be 40% faster, taking 6.6 min (range 2.9–12.4) without instrument tracking and 4.0 min (1.9–6.3) with it. Radiation also decreased by 91% (2.52 vs. 0.23 mGy with instrument tracking). **Conclusions:** Experience significantly enhanced the accessibility of Kambin’s triangle, reducing time and radiation exposure by about two-thirds. Regardless of experience, instrument tracking reduced radiation exposure by 90%. Additionally, familiarizing PITs with instrument location can eliminate 70% of the learning curve for those inexperienced with accessing Kambin’s triangle.

## 1. Introduction

Occupational exposure to ionizing radiation remains an important health concern in surgical disciplines that rely on intraoperative imaging, such as orthopedic and spine surgery. Repeated exposure to X-rays, particularly during fluoroscopy-guided procedures, has been implicated in increasing the risk of malignancies and other adverse health outcomes among surgeons, including elevated rates of thyroid, breast, and head and neck cancers [1]. These risks have become increasingly relevant as the utilization of minimally invasive (MIS) techniques, requiring extended fluoroscopic guidance, continues to grow within surgical practice.

Despite occupational dose limits and the implementation of regulatory standards for medical professionals, studies indicate that those in certain surgical subspecialties experience higher cumulative radiation exposures than others, with spine and trauma surgeons at particularly increased risk [2,3]. Modern protective strategies, such as lead shielding, pulsed and low-dose fluoroscopy, distance optimization, and procedural enhancements, have been developed and widely endorsed to reduce intraoperative X-ray exposure for both patients and surgical staff [4]. However, the adoption and practical efficacy of these techniques can vary by setting and surgical approach, leading to ongoing concern regarding cumulative occupational exposure.

Endoscopic spine surgery (ESS) marks a paradigm shift from traditional open approaches to an ultra-minimally invasive procedure. ESS utilizes a small incision with limited muscle dissection and the direct visualization of neural elements [5]. ESS has been widely proven to be equivalent to open or traditional approaches for laminectomy and discectomy with a reduced length of stay and total adverse events [6,7,8]. It demonstrates reduced infection rates and opioid use and lower visualized analog scale (VAS) scores compared to open surgery, making it a superior choice for many patients [9,10,11].

Despite its multiple advantages, ESS still carries a heightened risk of radiation exposure for operating room staff and patients compared to a traditional open approach. ESS most commonly utilizes fluoroscopy to provide real-time two-dimensional guidance for instrument placement and trajectory. Fluoroscopy remains the most prevalent method due to familiarity and speed; some surgeons have transitioned to the use of computed tomography (CT) imaging [12,13,14]. While this may reduce the risk of radiation exposure for operating staff, it carries a burden of time and radiation exposure for patients with an average radiation exposure of 1–31 mSv [15].

In ESS utilizing C-arm fluoroscopy, evidence suggests that procedural X-ray exposure for surgeons averages approximately 1.5–2.1 mSv per operation, an amount that generally remains well below established annual safety limits [16]. Nevertheless, the increasing procedural frequency and evolving practice patterns highlight the need to continually assess and optimize radiation safety.

One recent advanced instrument navigation technology is optical 2D fluoroscopic-based navigation, which is capable of decreasing physician, operating room (OR) staff, and patient radiation exposure. This platform utilizes intraoperative fluoroscopy for real-time live fluoroscopic guidance [17]. It features optical navigational fiducials on a low-profile frame linked to the C-arm fluoroscope’s radiation source and camera. Adaptable navigational arrays placed on existing instruments allow for the real-time tracking of their position relative to the fluoroscope. After initial calibration and registration images are acquired, this system allows for real-time digital instrument tracking in two dimensions (AP and lateral fluoroscopic image) without the need for any further images to be taken [17]. With the implementation of this technology, radiation exposure may be significantly reduced without compromises in anatomical accuracy [18]. This platform has been validated for pedicle screw placement and endoscopic transforaminal lumbar interbody fusion (TLIF) [19]. However, its application in endoscopic localization to Kambin’s triangle has not been thoroughly studied [20], with only a few studies comparing TrackX to conventional fluoroscopy [17,20,21,22,23] and none studying physicians in training (PITs). This study aims to highlight the use of 2D fluoroscopic navigation as a new adjunctive image guidance technique to reduce radiation exposure in surgeons, OR staff, and patients without compromising accuracy. To our knowledge, this is the first study assessing the usage of this 2D fluoroscopic navigation platform among PITs in a nonoperative setting.

## 2. Materials and Methods

### 2.1. Study Design and Participants

This study was designed as a pilot feasibility/exploratory experiment with a prospective assessment of the efficacy of 2D fluoroscopic optical navigation (TrackX Version 2, Hillsborough, NC, USA) compared to conventional fluoroscopic guidance during lumbar endoscopic operations. No sample size was calculated for PIT inclusion. Seven PITs were recruited from a single institution for testing, spanning from medical student (postgraduate year [PGY] 0) to residency (PGY 7). Participants were selected based on availability during a single scheduled training session when the TrackX system was accessible, and their prior endoscopic experience varied from none to more than 60 cases. We kept track of demographic characteristics, including PGY status, the number of endoscopic cases performed prior, and the spinal levels they had experience with.

### 2.2. Procedure Task

**Figure 1:**
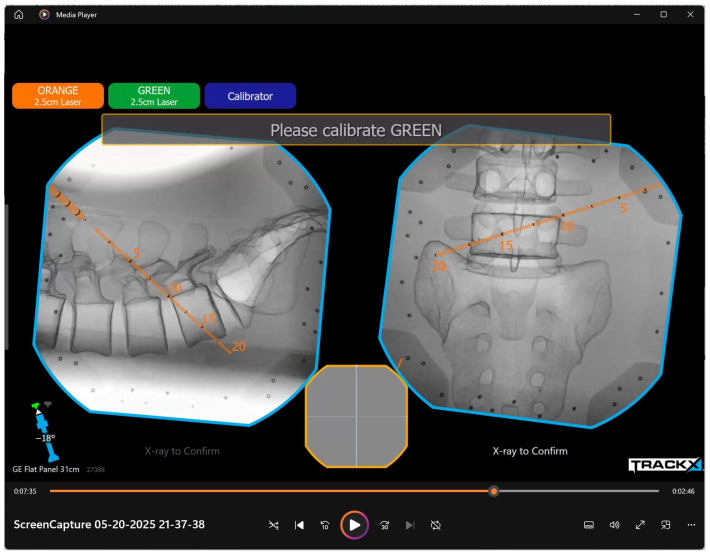
A screen capture of the TrackX interface demonstrating live localization in an AP and lateral view. The green tracker was not in use.

Each PIT executed standardized lumbar transforaminal endoscopic access procedures under two conditions: (1) conventional fluoroscopic guidance and (2) TrackX navigation (Figure 1). Every individual executed both conditions at spinal levels spanning from L1/2 to L5/S1. PITs were randomly split into two ORs and given a spinal level from L1/2 to L5/S1 and a side at random. Both ORs had the same sawbone models embedded in a gel cast secured to a standard radiolucent operating table using tape.

**Figure 2:**
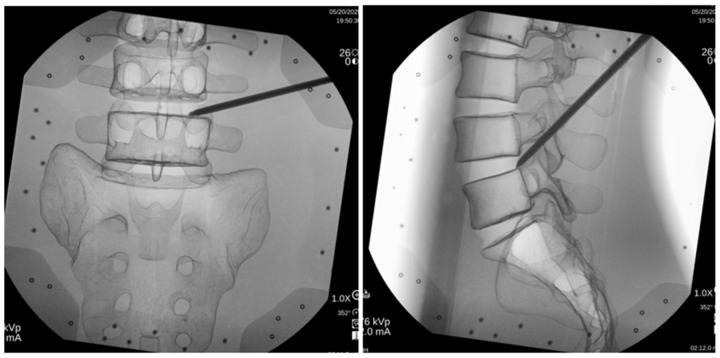
Confirmatory fluoroscopic AP (**left**) and lateral (**right**) views correlating with TrackX live tracking of Figure 1.

There was a C-arm in each OR, but only one of them also had the 2D optical navigation system in place. To eliminate bias, PITs were randomly given an OR to start with, but the randomization was based on OR order, spinal level, and approach side to minimize order effects. PITs were instructed to mark their entry target on the skin of the sawbone model. After that, they were able to use a primary dilator to Kambin’s triangle to continue with localization. The formal documentation of prior TrackX exposure was not collected; however, given the early adoption phase of the technology at our institution, all participants had minimal prior exposure (estimated fewer than ten cases). The time for both sets was recorded, as well as the radiation dose for each participant (Figure 2).

### 2.3. Outcome Measures

The main outcomes were procedure time and radiation exposure, which were split into two phases: the skin phase, which consisted of the time and radiation needed to progress from skin to the first bony contact, and the targeting phase, which consisted of the time and radiation needed to reach the final target location. The total time of the process and the total amount of radiation exposure were both found by adding together the times of both phases.

### 2.4. Consistency and Frequency of Advantage

We used the standard deviation (SD) for both procedure time and radiation exposure under each scenario to see how performance changed among PITs. The ratio of variability between conventional fluoroscopy and TrackX was used to show relative consistency. To measure how reproducible the TrackX advantage was, we counted how often TrackX performed better than conventional fluoroscopy in individual trials and presented the results as fold differences (for example, “10× lower radiation”).

### 2.5. Stratification by Experience

Additionally, PITs were divided into two groups based on prior endoscopic exposure/experience: a low-experience (LE) group (≤20 cases) and a high-experience (HE) group (≥30 cases) based on the self-reported number of endoscopic exposures. We compared conventional fluoroscopy and TrackX within each group using the formula 1 − (high-experience/low-experience) to determine whether prior experience impacted performance.

### 2.6. Statistical Analysis

The data were collected and analyzed using Excel and STATA (version 17). All continuous data are shown as the mean ± SD. Because a prior power analysis was not conducted before study initiation, post hoc power analyses were performed for the primary paired endpoints using a paired t-test design. To find the percent reduction, we used the following formula: (conventional − TrackX) ÷ conventional × 100. We conducted correlation analyses using linear regression to look at the associations between (i) procedure time and prior endoscopic experience, (ii) radiation exposure and prior endoscopic experience, (iii) procedure time and PGY level, and (iv) radiation exposure and PGY level during conventional fluoroscopy and TrackX navigation. A *p* value < 0.05 was considered significant.

## 3. Results

### 3.1. Background Characteristics

Seven PITs, ranging from postgraduate year (PGY) 0 to PGY 7 and with prior endoscopic experience from 0 to 60 cases, performed lumbar endoscopic tasks at spinal levels L1–2 through L5/S1 using both conventional fluoroscopic guidance and the TrackX navigation system. The endoscopic experiences of participants were different across various training levels. PITs in PGY 0–1 had no previous experience. One PGY 2 was exposed to 10 cases prior, and one PGY 4 was exposed to 30 cases. One of the two PGY-6 participants was exposed to 60 cases, and the other had worked on 20 cases. The one PGY 7 included in this study also had 30 prior endoscopic exposures (Table 1).

### 3.2. Procedure Time and Radiation Exposure

The cumulative procedure time across all participants was 2777 s with conventional guidance compared with 1678 s with TrackX, yielding a 40% overall reduction in total operative time. The mean total time per case decreased from 396.7 ± 38.0 s to 239.7 ± 46.5 s. Reductions were observed in both phases: skin time decreased by 42% (138.7 s to 81.1 s) and targeting time by 39% (258.0 s to 158.6 s). Radiation exposure was markedly lower with TrackX, with cumulative radiation decreasing from 17.62 mGy to 1.60 mGy, an overall 91% reduction. The average radiation per procedure decreased from 2.52 ± 1.21 mGy with conventional guidance to 0.23 ± 0.16 mGy with TrackX. Phase-specific analysis showed a 100% reduction in skin-phase radiation (0.94 to 0 mGy) and an 86% reduction in targeting-phase radiation (1.57 to 0.23 mGy) (Table 2). The post hoc power analyses performed to estimate power for the primary endpoints demonstrated a lower power for procedure time (42.4%) compared to radiation exposure with an achieved power of 96.5%. This value reflects substantial variability in the procedure time, despite a mean reduction of 157 s. For radiation exposure, the higher power was consistent with a large effect size relative to within-subject variability. Furthermore, sample size estimation based on observed effect sizes demonstrated that 15 participants would be required to achieve 80% power for procedure time, whereas only 5 participants would be required for radiation exposure.

### 3.3. Consistency and Frequency

The standard deviation for targeting time was 217.1 s under conventional fluoroscopic guidance compared with 54.3 s with TrackX. Conventional tracking was 4.0 times more inconsistent for procedural time and 7.4 times more inconsistent for radiation exposure. Regarding frequency, TrackX outperformed conventional guidance with 10-fold lower radiation exposure, and there were procedure time improvements in most individual comparisons (0.65 times faster overall) (Table 2).

### 3.4. Impact of Endoscopic Experience

PITs were stratified by prior endoscopic experience into LE (≤20 prior cases; *n* = 4) and HE (≥30 prior cases; *n* = 3) groups (Table 3). Under conventional fluoroscopic guidance, the HE group demonstrated substantially faster procedure times and lower radiation exposure than the LE group, and the mean total time decreased from 543.3 s in the LE group to 201.3 s in the HE group, a 63% reduction. Radiation exposure similarly fell from 3.5 mGy to 1.3 mGy (also 63% lower).

Among LE PITs, TrackX reduced procedure time from 543.3 s to 302.8 s and radiation from 3.5 mGy to 0.3 mGy, corresponding to a 91% reduction in radiation and a 44% reduction in time. High-experience PITs also benefited, with procedure time decreasing from 201.3 s to 155.7 s (23% faster) and radiation decreasing from 1.3 mGy to 0.2 mGy (85% lower). Although experience independently improved performance, conventional fluoroscopic guidance still exposed experienced PITs to 88% more radiation than TrackX. Notably, the initial use of TrackX allowed inexperienced participants to achieve approximately 70% of the performance benefit, with a 4 min (240.5 s) reduction in performance time, whereas experienced participants had a 45.6 s reduction in performance time.

### 3.5. Correlation Analysis

**Figure 3:**
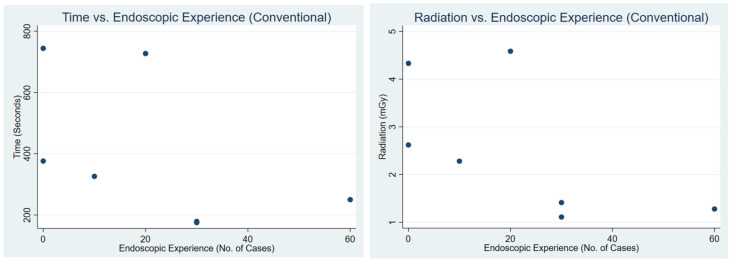
This figure shows a scatterplot of time vs. endoscopic experience and radiation vs. endoscopic experience during conventional fluoroscopic use.

**Figure 4:**
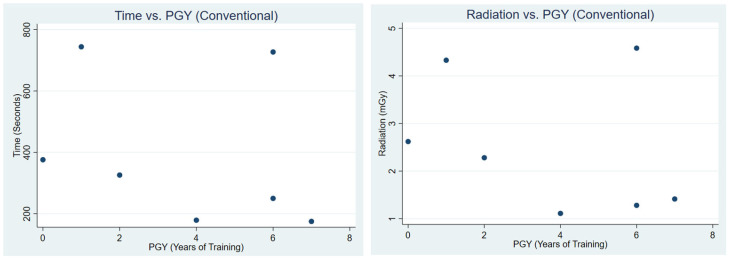
This figure illustrates a scatterplot of time vs. PGY and radiation vs. PGY during conventional fluoroscopic use.

Correlation analysis demonstrated differing patterns between conventional guidance and TrackX navigation (Table 4). Under conventional guidance, both procedure time and radiation exposure showed a moderate negative correlation with prior endoscopic experience (R = −0.52, R^2^ = 0.27 for time; R = −0.61, R^2^ = 0.37 for radiation). In contrast, correlations with PGY level were weak for both time (R = −0.28, R^2^ = 0.08) and radiation (R = −0.30, R^2^ = 0.09). Figure 3 and Figure 4 present a scatterplot of procedural time and radiation vs. endoscopic experience and PGY, respectively.

**Figure 5:**
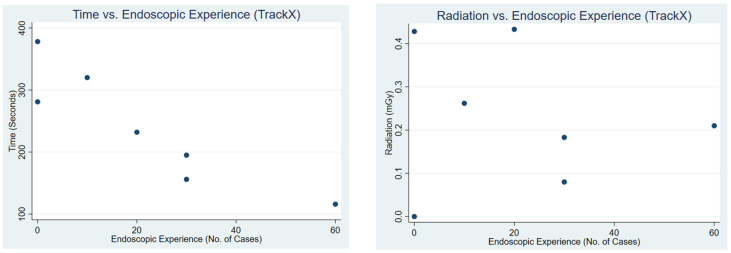
This figure presents a scatterplot of time vs. endoscopic experience and radiation vs. endoscopic experience during TrackX use.

**Figure 6:**
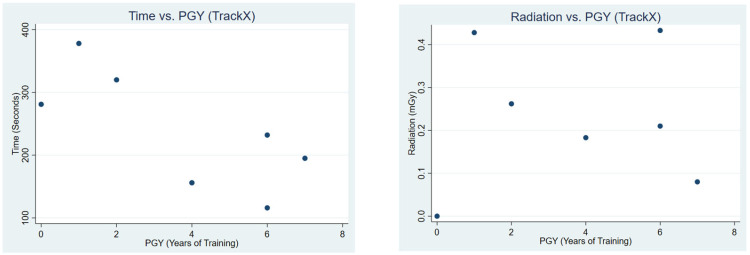
This figure presents a scatterplot of time vs. PGY and radiation vs. PGY during TrackX use.

With TrackX, the relationship between endoscopic experience and performance was more pronounced. Procedure time demonstrated a very strong correlation with prior endoscopic experience (R = −0.90; R^2^ = 0.81) (Figure 5). Conversely, radiation exposure was essentially uncorrelated with experience (R = −0.12; R^2^ = 0.01) or PGY (R = 0.06; R^2^ = 0.00). The correlation of procedure time with PGY was moderate (R = −0.74; R^2^ = 0.55) but weaker than that with direct case experience (Figure 6).

## 4. Discussion

To our knowledge this is the first study to evaluate the use of the TrackX navigation system compared with conventional fluoroscopic guidance for lumbar endoscopic procedures among PITs, with a focus on operative efficiency, radiation exposure, consistency, and the influence of prior endoscopic experience on PITs. This study demonstrates an 88% reduction in radiation exposure regardless of years of experience or exposure to TrackX technology with an increase in speed by 23% independent of previous endoscopic experience. With the implementation of instrument tracking, the surgeon’s radiation exposure can be significantly reduced during ESS without compromising accuracy. Instrument tracking also showed a clear benefit in new trainees with limited exposure to endoscopy, demonstrating it as an effective tool for new learners.

TrackX, first described by Wang et al. [21], in a 2019 feasibility study showcasing pseudo-live tracking [24], has been recognized for its ability to reduce procedure time in MIS lumbar procedures. Our study showed a 40% overall reduction in operative time when TrackX was used compared with conventional fluoroscopy. Similarly, Wang et al. showed an 81% cumulative time reduction with TrackX [17], and Hamouda et al. demonstrated that 4.21 min and 8.14 mGy radiation were saved by TrackX by placing an initial dilator into Kambin’s triangle in 23 patients [20]. This suggests that TrackX has multiple benefits: reduced operative time, improved efficiency of access to Kambin’s triangle, and minimized complications induced by localization. This remains valid even in advanced cervical ESS over CT-guided navigation systems [25]. Importantly, procedure time reductions were not confined to highly experienced PITs in this study, with less experienced PITs also demonstrating substantial time savings with TrackX, suggesting that the system provides an equalizing effect that helps bridge the performance gap between novices and experts. This is an important finding given the steep learning curve ascribed to transforaminal endoscopic spine surgery [5].

A recent meta-analysis by Cristofalo et al. [3], reviewing 34,744 physicians, demonstrated an increased risk of breast cancer among female physicians exposed to ionizing radiation. Despite best radiation exposure practices, the risk of cancer in surgeons remains elevated compared to non-surgeon colleagues [2,3,26]. In spine surgery, fluoroscopy-guided spinal procedures involve a notable amount of radiation exposure [17]. Rampersaud et al. [27] and Mariscalco et al. [28] reported a 12-fold and 20-fold increase in radiation exposure with fluoroscopically assisted pedicle screw insertion and MIS lumbar discectomy, respectively. Although Airo CT and O-Arm were designed to minimize radiation exposure for surgeons and OR staff during MIS instrumentation [29], they may increase radiation exposure for patients, decrease surgeon efficiency and slow the workflow [17,30]. However, consistent with Wang et al. [17], TrackX has been shown to reduce radiation exposure significantly, with a 91% overall reduction and average radiation per procedure dropping tenfold compared with conventional fluoroscopy in this study. Although skin-phase radiation was drastically reduced, it is noteworthy that this measurement did not include the potential skin-phase radiation exposure from the device during the initial acquisition of images for registration. Targeting-phase radiation was reduced by 86%. The critical implications of these findings underscore the ability of this type of navigation system to significantly enhance radiation safety while maintaining procedural accuracy [21,23]. In particular, studies have shown that with the utilization of instrument tracking, the average radiation exposure was significantly lower than the reported averages for transforaminal endoscopic surgery [16,17] and 100-fold lower than the potential radiation exposure for patients when utilizing O-Arm navigation [15].

The steep learning curve in ESS when coupled with conventional fluoroscopy is well documented [5]. In this study, we demonstrated that prior endoscopic experience strongly influenced outcomes under conventional fluoroscopic guidance, with high-experience PITs demonstrating markedly faster procedure times and lower radiation exposure compared with their less experienced counterparts; however, correlations with PGY level were weak, suggesting that formal years of training alone were not sufficient predictors of efficiency in fluoroscopic-guided procedures. Although TrackX utilization itself has a learning curve, studies have demonstrated superior pathology localization and rapid improvement in the learning curve in various lumbar discectomy [17,23].

Additionally, our results showed that TrackX reduced the experience gap, with a significant reduction in radiation exposure and lower procedure time for less experienced PITs, which allowed them to approach the performance levels of experienced participants. Even for high-experience PITs, TrackX still conferred benefits, with a reduction in both radiation and procedure time, suggesting the dual role of TrackX, including accelerating the learning curve for novices while still enhancing efficiency and safety for experts. Particularly noteworthy is the fact that inexperienced PITs, using TrackX for the first time, achieved approximately 70% of the performance benefit with a 4 min (240.5 s) reduction in performance time in inexperienced participants compared to an only 45.6 s reduction in performance time for experienced participants. Although this performance improvement was substantial, the small sample size and limited number of tracked attempts restrict the ability of this study to accurately characterize the PIT learning curve, and the findings may be interpreted as demonstrating performance differences across experience levels. Additionally, although the gel-embedded sawbone model allowed for procedural standardization, it may not fully replicate clinical scenarios, including soft tissue resistance, bleeding, patient discomfort, anatomical variability, and the complexity of the operative workflow. Despite these limitations, the evidence suggests that 2D fluoroscopic navigation systems may democratize access to efficient and safe procedures, reducing dependence on long-term cumulative experience. Thus, navigation with TrackX may facilitate adoption among trainees, who are likely to use ESS in their future practices given its expanding popularity and broadening indications [5,31]. Also, the accurate and precise virtual projection of surgical instruments with TrackX facilitates the identification of midline and proper trajectories during discectomies and implant placement [18,22]. A prior study reported that the learning curve is initially slower but improves after approximately 11 cases, after which operative time becomes consistently shorter [23]. In a clinical series, Wang et al. reported 96.4% screw placement accuracy in 24 patients, with acceptable overall accuracy but variable complication risks ranging from asymptomatic screw breach to limb weakness and radiculopathy during multilevel lumbar fusion [18]. Other reports have demonstrated no adverse patient outcomes [17], suggesting that as the learning curve plateaus, TrackX navigation may be clinically effective in improving operative efficiency and intraoperative safety [17,22].

As hypothesized, radiation exposure was decoupled from PGY levels in this study. This suggests that TrackX standardizes radiation safety across all levels of experience, attenuating one of the most concerning disparities between novices and experts. The moderate correlation of PGY with procedure time under TrackX indicates that surgical maturity still plays a role but less so than direct case experience. Together, these findings imply that TrackX shifts the determinants of procedural efficiency from radiation safety toward technical refinement, with experience remaining important for speed but less so for safety.

This study was limited by a small number of participants and the single-institution setting, which may limit generalizability. However, every possibility was taken to reduce bias with the randomization of OR order, operative level, right versus left, and strict timing. An optimal follow-up study could include both residents and young endoscopic spine surgeons utilizing cadaveric specimens and multiple levels per participant. Post hoc power analyses also demonstrated that this study was underpowered for procedure time but adequately powered for radiation exposure. Additionally, this study did not assess the long-term retention of radiation safety practices or the impact of navigation technology on clinical outcomes beyond radiation exposure and procedure time. Therefore, the findings should be interpreted cautiously, as the intent was exploratory rather than providing definitive evidence of the efficacy of the 2D fluoroscopic navigation system (TrackX). Also, the experimental design using a gel-embedded sawbone model may not fully reproduce clinical conditions, including patient anatomy, soft tissue handling, and intraoperative workflow. However, most studies on TrackX in the existing literature have primarily focused on instrument tracking accuracy, intraoperative workflow, safety, and learning curves, with limited attention to patient-specific factors such as anatomical variability and soft tissue characteristics. This gap underscores the need for further research to better define the performance of TrackX across diverse patient populations. Future research should also explore the scalability of these findings across diverse training environments and endoscopic procedures, as well as the cost-effectiveness and learning curve associated with navigation system adoption.

## 5. Conclusions

In this pilot exploratory study, we observed that the addition of optical 2D fluoroscopic navigation technology can reduce radiation exposure in PITs or experienced surgeons while accessing Kambin’s triangle for ESS. These preliminary findings support 2D fluoroscopic navigation technology as a tool for reducing radiation exposure in novice endoscopic spine surgeons while continuing to show benefits in time efficiency and radiation exposure for more experienced surgeons. This study is limited by a small sample size, and additional studies will need to be conducted to demonstrate the power of this approach. Larger clinical studies are also warranted to further evaluate the clinical efficacy of this instrument-tracking system. However, these results expose a promising new technology in reducing the risk of radiation for both the patient and the surgeon in ESS.

## Figures and Tables

**Table 1:** Descriptive characteristics and procedural parameters.

				Conventional				TrackX			
				Skin		Targeting		Skin		Targeting	
	PGY	No. of Endoscopic Surgeries	Spinal Levels	s	Rad	s	Rad	s	Rad	s	Rad
PIT 1	1	0	L5/S1	156	1.39	588	2.94	150	0	228	0.428
PIT 2	2	10	L4/5	165	1.551	161	0.73	102	0	218	0.262
PIT 3	6	60	L3/4	118	0.595	132	0.685	30	0	86	0.21
PIT 4	4	30	L2/3	130	0.798	49	0.313	57	0	99	0.183
PIT 5	7	30	L1/2	68	0.569	107	0.845	44	0	151	0.08
PIT 6	6	20	L5/S1	184	1.326	543	3.258	54	0	178	0.433
PIT 7	0	0	L4/5	150	0.382	226	2.239	131	0	150	0

PGY, postgraduation year; PGY 0 = medical student; s, seconds; Rad, radiation; PIT, physician in training.

**Table 2:** Differences between conventional guidance and TrackX performance.

Variables		Conventional	TrackX	Absolute Difference	% Improvement with TrackX
Procedure time *					
	Total s	2777.00	1678.00	1099.00	40%
	Total rad	17.62	1.60	16.02	91%
Procedure performance					
Total procedure time, s					
	Skin PT, s	138.71	81.14	57.57	42%
	Targeting PT, s	258.00	158.57	99.43	39%
Total radiation exposure, rad					
	Skin PT, s	0.94	0	0.94	100%
	Targeting PT, s	1.57	0.23	1.34	86%
Consistency (Variability) ^‡^					
	SD of TT, s	4.0× inconsistent			
	SD of TR, rad	7.4× inconsistent			
Frequency of advantage					
	TT		0.65× faster		
	TR		10.04× lower		

* Combined skin and targeting phase sec and rad. ^‡^ Consistency showed relative reduction in variability. Frequency of advantage was estimated with formula 1/(1 − TrackX total sec) − 1 for sec and 1/(1 − TrackX total rad) − 1 for rad. SD, standard deviation; Sec, seconds; Rad, radiation; PT, phase time; TT, total time; TR, total radiation.

**Table 3:** Physicians in training grouped by PGY endoscopic experience.

Metrics		LE (<20 Cases)	HE (>30 Cases)	Between-Experience Group Difference
No. of Participants		4	3	
Total Time (s)				
	Conventional	543.3	201.3	63%
	TrackX	302.8	155.7	49%
Between-Procedure Group Difference		44%	23%	
Radiation (rad) *				
	Conventional	3.5	1.3	63%
	TrackX	0.3	0.2	44%
Between-Procedure Group Difference		91%	85%	

* The risk of radiation exposure was estimated using the formula 1 − (average TrackX rad/average conventional rad): 1 − (0.2/1.3) = 88%. Also, the effect of TrackX use on improving experience performance was estimated using the equation (mean Conventional_LE_ − TrackX sec_LE_)/(mean Conventional_LE_ − Conventional_HE_) = (543.3 − 302.8)/(543.3 − 201.3) = 70%. PGY, postgraduation year; LE, low experience; HE, high experience.

**Table 4:** Correlation analysis comparing time and radiation with endoscopic experience and PGY between procedural groups.

Procedural Method	Outcome Variable vs. Predictor Variable	Model Fit; R (R-Squared)	β (SE)	95% CI	*p* Value
**Conventional**					
	Time vs. Endoscopic Experience	−0.52 (0.27)	0.003 (0.011)	3.3 × 10^−8^–191.02	0.229
	Radiation vs. Endoscopic Experience	−0.61 (0.37)	0.960 (0.023)	0.902–1.021	0.148
	Time vs. PGY	−0.28 (0.08)	2.16 × 10^−11^ (8.19 × 10^−10^)	1.13 × 10^−53^–4.14 × 10^31^	0.545
	Radiation vs. PGY	−0.30 (0.09)	0.857 (0.191)	0.483–1.518	0.518
**TrackX**					
	Time vs. Endoscopic Experience	−0.90 (0.81)	0.019 (0.016)	0.002–0.168	0.005
	Radiation vs. Endoscopic Experience	−0.12 (0.01)	0.999 (0.003)	0.990–1.008	0.802
	Time vs. PGY	−0.74 (0.55)	1.33 × 10^−11^ (1.35 × 10^−10^)	7.11 × 10^−23^–2.499	0.056
	Radiation vs. PGY	0.06 (0.00)	1.003 (0.027)	0.938–1.074	0.890

*p* value: <0.05 means significant. PGY, postgraduation year; β (SE), coefficient (standard error); CI, confidence interval.

## Data Availability

The raw data supporting the conclusions of this article will be made available by the authors on request.

## Abbreviations

The following abbreviations are used in this manuscript:

PITs	Physicians in training
HE	High exposure
LE	Low exposure
MIS	Minimally invasive
ESS	Endoscopic spine surgery
VAS	Visualized analog scale
CT	Computed tomography
OR	Operating room
TLIF	Transforaminal lumbar interbody fusion
PGY	Postgraduate year
SD	Standard deviation
